# Patient Experience with a Gynecologic Oncology-Initiated Genetic Testing Model for Women with Tubo-Ovarian Cancer

**DOI:** 10.3390/curroncol29050288

**Published:** 2022-05-15

**Authors:** Michaela Bercovitch Sadinsky, Joanne Power, Enza Ambrosio, Laura Palma, Xing Zeng, William D. Foulkes, Evan Weber

**Affiliations:** 1 Department of Human Genetics, McGill University, Montreal, QC H3A0C7, Canada; laura.palma@muhc.mcgill.ca (L.P.); william.foulkes@mcgill.ca (W.D.F.); evan.weber@muhc.mcgill.ca (E.W.); 2 Division of Medical Genetics, Jewish General Hospital, McGill University, Montreal, QC H3T1E2, Canada; 3 Division of Gynecologic Oncology, McGill University Health Centre, Montreal, QC H4A3J1, Canada; joanne.power@muhc.mcgill.ca (J.P.); enza.ambrosio@muhc.mcgill.ca (E.A.); xing.zeng@mcgill.ca (X.Z.); 4 Division of Medical Genetics, McGill University Health Centre, Montreal, QC H4A3J1, Canada; 5 Department of Specialized Medicine, Lady David Institute, Jewish General Hospital, McGill University, Montreal, QC H3T1E2, Canada; 6 Cancer Research Program, Research Institute of the McGill University Health Centre, McGill University, Montreal, QC H4A3J1, Canada

**Keywords:** hereditary cancer, genetic counselling, genetic testing, patient experience, mainstreaming

## Abstract

Background: Up to 20% of women diagnosed with tubo-ovarian carcinoma carry a germline pathogenic variant in a cancer-predisposing gene (e.g., *BRCA1/BRCA2*). Identifying these variants can help to inform eligibility for therapies, guide surveillance and prevention of new primary cancers, and assess risk to family members. The Gynecologic Oncology-Initiated Genetic Testing Model (GOIGT) was initiated at the McGill University Health Centre (MUHC) to streamline universal germline genetic testing for this population, while addressing the limited resources in the public healthcare system. This study aimed to evaluate the patient experience of participating in this model. Methods: Study participants were patients diagnosed with high-grade non-mucinous epithelial tubo-ovarian cancer who underwent genetic testing through the GOIGT model between 1 January 2017 and 31 December 2020. Eligible participants completed the retrospective questionnaires at least one month after result disclosure. Results: A total of 126 patients were tested through the GOIGT model during the study period, of which 56 were invited to participate. Thirty-four participants returned the study questionnaire. Overall, participants did not report decision regret following the genetic testing and were satisfied with the GOIGT model. Participants reported low levels of uncertainty and distress related to the implications of their test results for themselves and their family members. Conclusions: The results of this study support the continued implementation of mainstreamed genetic testing models for women with high-grade non-mucinous tubo-ovarian cancer. Further studies are required to compare experiences for patients with different genetic test results.

## 1. Introduction

High-grade serous carcinoma of the ovary, fallopian tube, or peritoneum (HGSOC) is the leading cause of gynecologic cancer-related deaths in women [1]. Up to 20% of women with tubo-ovarian carcinoma carry germline mutations in a growing number of susceptibility genes (e.g., *BRCA1, BRCA2, RAD51C, RAD51D,* and *BRIP1*) [2,3,4,5]. Germline genetic testing of patients with tubo-ovarian cancer can provide valuable information regarding treatment options and future cancer risks, as well as facilitate risk assessment for family members who may benefit from increased cancer surveillance or risk reduction methods [6,7]. In 2017, the Society of Gynecologic Oncology of Canada (GOC) released a position statement, “No Woman Left Behind”, whereby they posit that all women with high-grade serous ovarian, tubal, or peritoneal carcinoma should undergo *BRCA1/2* testing [8,9]. 

A study of women diagnosed with HGSOC in Ontario between 1997 and 2011 found that only 6.6% of patients were seen for a genetics consultation within two years of their initial diagnoses, despite increased access to genetic testing in healthcare [10]. At the McGill University Health Centre (MUHC), Montreal, QC, a retrospective review of women diagnosed with HGSOC from January 2008 to July 2017 found that only 50.9% of women were referred to the Division of Medical Genetics for genetic testing [11]. 

Streamlining the genetic testing process to increase access to testing is commonly referred to in the literature as mainstreamed genetic testing. Under this collaborative model, pre-test counselling, consent, and genetic test coordination is typically performed by oncologists or oncology nurses, while result disclosure, post-test counselling, and follow-up is facilitated by geneticists or genetic counsellors. These protocols increase the proportion of patients accessing genetic testing for these patient populations, while reducing the delay from cancer diagnosis to genetic test result disclosure [12,13,14,15]. Similar projects have been successful for oncology-based genetic testing for patients with breast cancer, with overall high patient satisfaction [16,17,18]. 

Prior evaluations of these mainstreaming protocols demonstrate an increase in the number of patients tested, a reduction in overall turnaround time, and an overall positive self-reported patient experience. McLeavy and colleagues [19] found that patients with ovarian cancer who underwent mainstreamed genetic testing for the *BRCA1* and *BRCA2* genes reported low levels of decision regret, uncertainty, or lack of control following their test. Likewise, the ENGAGE study [20], an international study of an oncology-based testing model in patients with ovarian cancer, identified an overall positive experience for patients throughout the testing process and after the results disclosure. Patients cited acquiring knowledge for their children’s benefit as their main motivation for participating in genetic testing; however, as connections between therapy success and specific pathogenic variants are identified, such as the potential for poly ADP ribose polymerase inhibitor (PARPi) treatment for ovarian cancer in patients who carry a germline *BRCA1/2* pathogenic variant, these motivations may shift [20]. 

**Figure 1:**
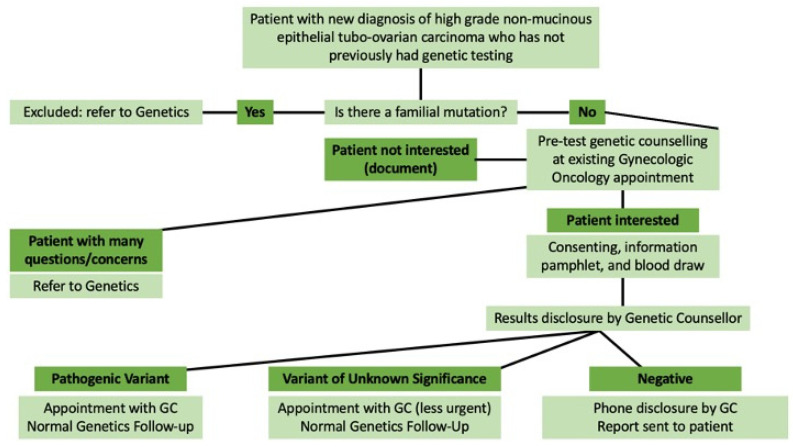
Gynecologic Oncology-Initiated Genetic Testing (GOIGT) Model.

In August 2017, the MUHC introduced an oncology-based genetic testing model for women with HGSOC in collaboration with Medical Genetics and Gynecological Oncology, referred to as the Gynecologic Oncology-Initiated Genetic Testing (GOIGT) model. In 2020, the protocol was expanded to include all patients with high-grade epithelial non-mucinous tubo-ovarian cancer. In this model, basic pre-test genetic counselling is provided by an oncologist after cytoreductive surgery so as not to overwhelm patients with additional information at the time of initial diagnosis or coordination of treatment. Gynecologic oncologists received training for key counselling points involved in pre-test counselling and were provided with a checklist of key points to be discussed with patients. This pre-test counselling was supplemented with an information pamphlet about genetic testing for ovarian cancer, possible results of testing, and implications of each result for themselves and at-risk family members. All patients were offered the opportunity to speak with a genetic counsellor in the case of additional questions, with zero uptake. Patients are then offered multi-gene panel testing through a commercial laboratory, with results disclosed by a genetic counsellor (Figure 1) [11]. A preliminary evaluation of the first year of this protocol showed a significant increase in genetic testing rates from 51% to 86% for this patient group and a significant decrease in the median time from cancer diagnosis to results disclosure, from 186 to 58 days [11]. Since the first year of the introduction of the GOIGT model, the genetic testing rate has increased to 98% (unpublished data). 

Building on the early success of the streamlined genetic testing model at our institution, the present study aimed to assess whether patients were satisfied with this novel model of care. Our objective was to evaluate the patient experience of a gynecologic oncologist-initiated genetic testing model at a single high-volume academic center. We performed a retrospective quantitative analysis of patient experience to evaluate patient satisfaction, distress, uncertainty, and levels of decision regret for the patients. Secondary outcomes included patient perception of the model itself, including areas for potential improvement to better address patient needs. 

## 2. Materials and Methods

### 2.1. Study Participants

The study was approved by the MUHC Research Ethics Board (2021-6562). A total of 126 women diagnosed with high-grade non-mucinous tubo-ovarian cancer were tested via a mainstreamed genetic testing pathway at a tertiary healthcare centre in Montreal, Quebec between August 2017 and December 2020. All patients were tested with a baseline panel including *BRCA1, BRCA2, BRIP1, CHEK2, PALB2, RAD51C, RAD51D,* and *TP53*. In November 2017, the mismatch repair genes were added to the panel (*MLH1, MSH2, MSH6, PMS2,* and *EPCAM*). In April 2020, the *ATM* gene was added. No age or family history restrictions were applied. Genetic testing was coordinated through the Gynecologic Oncology division, whereby oncologists received resources and training for pre-test counselling from the division of Medical Genetics. Post-test counselling was provided by telephone, videoconference, or in person by one of three cancer genetic counsellors at the centre. After the onset of the COVID-19 pandemic in March 2020, all post-test counselling was provided by telephone or videoconference. 

Individuals eligible to participate in the study were adult women who underwent genetic testing through the GOIGT protocol, had adequate fluency in English or French, and were able and willing to provide consent. Of the 126 women who participated in GOIGT, 23 patients were deceased at the time of study initiation. Three patients were excluded because their genetic test results were disclosed to another family member due to patient cognitive status, while one patient was unable to be reached for her result. An additional 12 patients were excluded due to the severity of their disease progression and overall poor health status at the time of recruitment. A remaining 12 patients were ineligible due to inadequate fluency in either English or French. One patient was omitted due to human error. 

**Figure 2:**
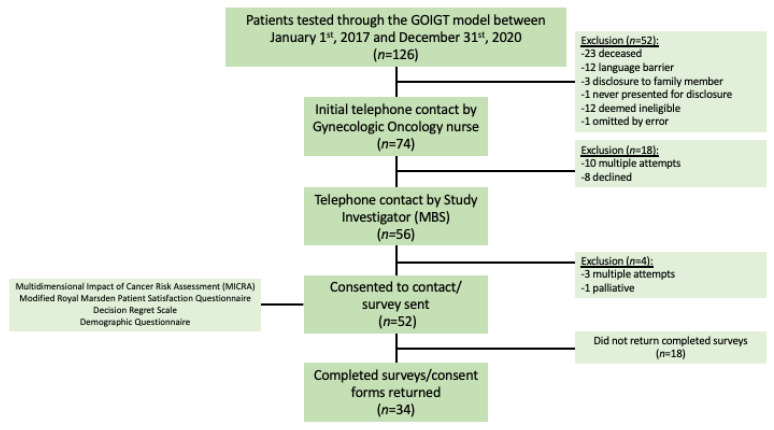
Study design and ascertainment of participants.

Seventy-four eligible patients were contacted by a member of the division of Gynecologic Oncology at least one month following disclosure of their genetic test results to gauge interest in participation. The earliest result disclosure took place in May 2018, approximately 2.5 years before the recruitment for this study began. Eight patients declined participation, while 10 patients could not be reached after multiple attempts. A total of 56 patients agreed to be contacted by the study investigator (MBS) to further discuss the study goals and requirements of whom 52 consented to receive the study materials. Three patients were unable to be reached by telephone and one patient had been transferred to palliative care and was deemed ineligible. All study materials including questionnaires and consent forms were sent to prospective participants by either mail or email. All women received a follow-up phone call from MBS to address any questions. Thirty-four respondents consented to participate and returned the study questionnaires, consistent with a 27% overall response rate and 46% response rate of those patients deemed eligible (Figure 2).

### 2.2. Measures

Participants completed four questionnaires as part of the survey package: a demographic questionnaire previously used by the Gynecological Oncology team [21], the Multidimensional Impact of Cancer Risk Assessment (MICRA) questionnaire, the Modified Royal Marsden Patient Satisfaction Questionnaire, and the Decision Regret Scale [20,22,23]. The questionnaires were identical for participants regardless of their genetic test results, and the study coordinator was blinded to participants’ genetic status. The demographic questionnaire and MICRA questionnaires were previously translated and validated in French. Approval and a license for the use of the French and English versions of the MICRA questionnaire were obtained from FACIT. With permission from the original authors, our group translated the Modified Royal Marsden Patient Satisfaction Questionnaire and Decision Regret Scale into French through forward and back translation. 

Participants selected “never”, “rarely”, “sometimes”, or “often” for each question on the MICRA. The questions included in the MICRA questionnaire were assigned into three subscales: distress, uncertainty, and positive experience. Reverse scoring was performed for those questions assigned to the positive experience subscale. A sum of the total score for each subscale was obtained and multiplied by the number of items in the subscale. This product was then divided by the number of items answered to produce the score for the respective subscale. The sum of the subscale scores produced the total MICRA score. 

The modified Royal Marsden questionnaire included a combination of 5-point Likert-scale questions, as well as yes or no questions. The Decision Regret Scale included five 5-point Likert-scale questions. Questions 2 and 4 were reverse coded so that a higher score indicated a higher level of regret. Scores on the Decision Regret Scale were then converted to a 0-100 scale by subtracting 1 from each item and multiplying that number by 25. The average of the sum of the items produced the final score. A score of 0 is consistent with no regret, while a score of 100 means high regret. 

### 2.3. Data Analysis

Descriptive statistics were presented for both categorical and continuous variables. Categorical variables were summarized as frequencies with percentages, and continuous variables were described as means with standard deviations, as well as medians and ranges, when applicable. All summarized questionnaire means were calculated using the specified methods in the original validations of the questionnaires. The total number of responses for each question was noted as some participants omitted questions. 

The final question on the Modified Royal Marsden Patient Satisfaction questionnaire allows for free-text comments and was used to contextualize the participants’ responses. Quotations from participant responses were used to provide additional richness to the data. 

## 3. Results

### 3.1. Participant Demographics

Of the 126 patients who were tested through the GOIGT model, 74 were initially deemed eligible amongst whom 56 agreed to be contacted by the study investigator about the study. Thirty-four participants (34/56, 60.7%) returned the study questionnaire. The mean age of diagnosis of tubo-ovarian cancer amongst study respondents was 64.6 years (standard deviation = 9.4 years). The majority of participants spoke English (20/34, 58.8%) or French (12/34, 35.3%) as their first language, with remaining participants identifying as either bilingual or citing a different primary language. The vast majority of respondents (30/34; 88.2%) reported living in an urban area, with 17.6% (6/34) of participants citing that they lived alone. Half of all respondents were retired, while 14.7% (5/34) of respondents reported being on disability or sick leave. The distribution of reported education levels was representative of what was seen in clinic at this tertiary healthcare centre (Table 1).

When comparing those participants who completed the study protocol and those who consented to receive a questionnaire but who ultimately did not return the materials, the median age was 66 (range 40–84) years of age and 61 (range 32–89) years of age, respectively. The median time in months between genetic test result disclosure and point of eligibility for this study (i.e., attainment of one-month post-result disclosure) was 17 (range 1–35) and 20 (range 1–38), respectively. Utilizing the Mann–Whitney test, there was no significant difference between the groups for age (*p* = 0.051) or time between genetic test result disclosure and point of eligibility for this study (*p* = 0.744). 

The genetic test results of respondents were reviewed after recruitment and data collection. Of note, 12/34 (35.3%) respondents carried a pathogenic variant, while two participants (0.59%) carried a variant of unknown significance (VUS). 

### 3.2. Multidimensional Impact of Cancer Risk Assessment

Overall, participants reported low “Distress” and “Uncertainty” scores, with moderate “Positive Experience” scores on the Multidimensional Impact of Cancer Risk Assessment questionnaire (Table 2). Seventy percent (23/33) of participants expressed worry about their personal risk of developing cancer in the future, and 41% (14/34) were frustrated about the lack of concrete prevention guidelines for their care. Eleven participants (11/34 = 32%) cited uncertainty about the meaning of their test results for their family members’ cancer risks. Approximately a quarter reported feeling sad or anxious about their genetic test results, while 41% (14/34) and 38% (13/34) reported feeling relieved or happy about their test result, respectively. Notably, the same 13 participants who reported feeling happy also reported feeling relieved by their result (13/34 = 38%). Upon review of their genetic test results, one patient had a positive result, one carried a VUS, and 11 had negative results. The additional participant who reported feeling relieved but not happy by their result had received a positive genetic test result.

### 3.3. Modified Royal Marsden Patient Satisfaction Questionnaire

**Figure 3:**
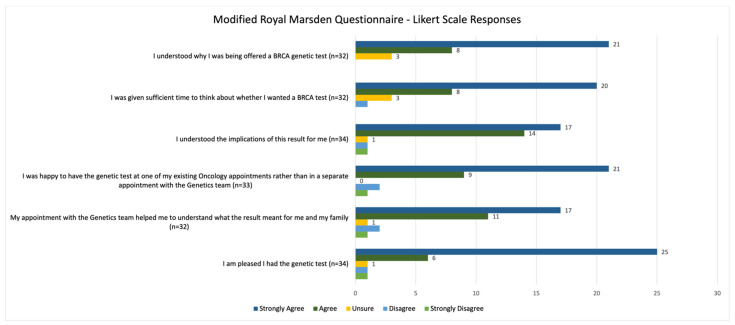
Summary of patient responses to Likert-scale questions from the modified Royal Marsden Patient Satisfaction questionnaire.

**Figure 4:**
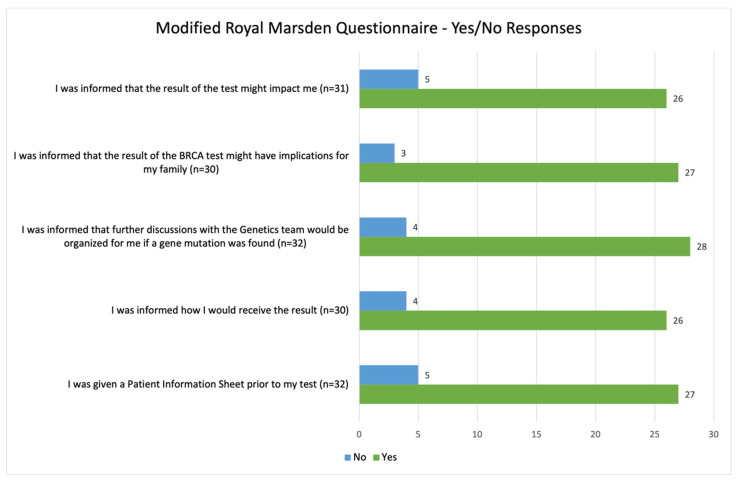
Summary of patient responses to the yes or no questions from the modified Royal Marsden Patient Satisfaction questionnaire.

The vast majority of participants (31/34) agreed or strongly agreed that they were pleased to have had genetic testing, while one participant was neutral, one participant indicated “disagree”, and one participant indicated “strongly disagree”. Ninety-one percent (31/34) of participants were happy to have had the genetic test at an existing oncology appointment, and 88% (28/32) agreed or strongly agreed that their post-test appointment with a genetic counsellor helped them to understand the implications of their test result for themselves and their family. Overall, participants cited a good understanding as to why they were offered testing as well as the implications of their test result for themselves (Figure 3 and Figure 4).

In the free-text responses to the questionnaire, two participants suggested that patients be offered a broader panel of genes included in the genetic test, especially for those who receive a negative result. One participant expressed that they “strongly believe that every cancer patient should have this test”, while multiple participants expressed appreciation for the efficiency, friendliness, and care provided by both the gynecologic oncology and genetics teams under this protocol. Notably, three participants explained that their genetic test was performed at a stressful and overwhelming time after receiving their initial cancer diagnosis, and that they benefitted from a family member or support person being present at their pre-test appointment to aid in the comprehension of the information. Likewise, participants expressed that the combination of their age and/or their treatment history may have impacted their memory of their experience with the GOIGT model (e.g., “chemo fog”). 

### 3.4. Decision Regret Scale

**Figure 5:**
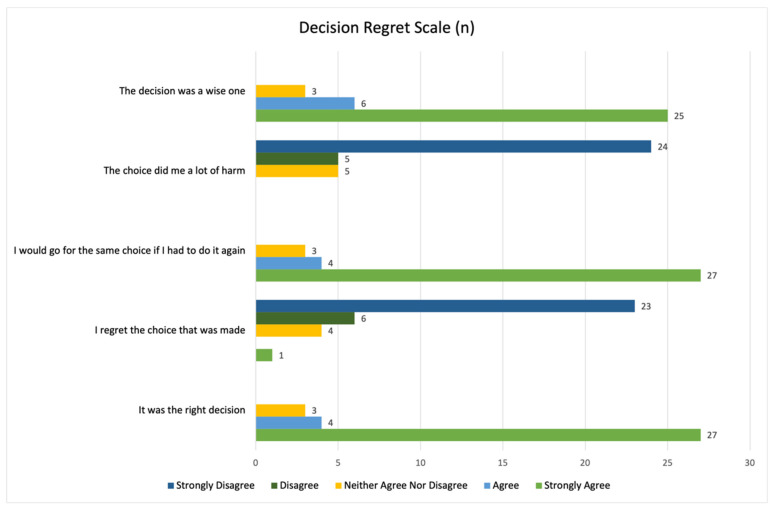
Summary of participant responses to the Decision Regret Scale.

A mean score of 14.56 (standard deviation = 15.34) on the Decision Regret Scale was cited by participants, with lower scores indicative of less decision regret. No participant disagreed or strongly disagreed that the decision to pursue genetic testing was the right choice, and only one participant regretted their choice to be tested. Likewise, all participants either agreed, strongly agreed, or provided a neutral response about whether they would make the same choice if they had make the decision again. None of the participants agreed or strongly agreed that the choice to have genetic testing harmed them (Figure 5).

## 4. Discussion

The GOIGT model of genetic testing for women with high-grade tubo-ovarian cancer was associated with overall high levels of patient satisfaction. Participants expressed satisfaction with the streamlined model for testing, preferring to have their pre-test counselling at an existing oncology appointment. This is supported by similar studies of mainstreamed genetic testing models [16,17,18,19,20]. Overall, participants felt that they received appropriate pre- and post-test counselling. Importantly, participants cited having a good understanding of the implications of possible genetic test results for themselves and their family members, suggesting that the informed consent process was not compromised by the streamlined testing model. Evidently, the oncology-initiated pre-test counselling was successful in transmitting the vital information that would typically be central to a traditional genetic counselling session. This further supports the current GOIGT model, and the multidisciplinary collaboration to make genetic testing more accessible to this vulnerable patient population. 

Previous research have shown that genetic testing rarely has negative psychosocial implications for patients with ovarian cancer, especially when there are implications for treatment or for other family members [18,19,24]. While our study was ongoing, McCuaig et al. [18] reported on the experience of patients with breast or ovarian cancer who experienced traditional genetic counsellor-mediated genetic testing and those who experienced oncologist-mediated testing at a different Canadian high-volume academic medical centre. Patients had similar MICRA scores regardless of their testing path. Although our study did not include a comparison group of patients receiving traditional genetic counselling, results from the current study further support these findings, with very limited negative psychosocial implications for patients undergoing genetic testing via the GOIGT model. Notably, in the previously mentioned study, patients who had a family history of cancer were more likely to have a lower positive experience score, highlighting the importance of post-test support for those with a family history of cancer to address any concerns of uncertainty or lack of closure, especially when disclosing a negative genetic test result. 

In the present study, 68% of respondents (23/33) expressed worry about their personal risk of developing cancer in the future, which has not been previously noted in the literature. Given that our cohort was not stratified based on genetic test results, we cannot differentiate or compare the responses between mutation carriers and non-carriers. Similarly, family history was not assessed in our study. Future comparison of our participants’ responses in the context of their family history can provide further insight into whether any adjustments to the GOIGT protocol are warranted, particularly for patients with uninformative test results. Likewise, this response may support increased clarification and discussion of the implications of each type of test result (positive, negative, VUS), and the resources available. 

Overall, self-reported decision regret was extremely low amongst participants. Decision regret refers to “a negative emotion associated with a reduction in satisfaction, quality of life, and physical health that results in a strained relationship between a patient and their healthcare provider” [25]. None of our participants reported that they disagreed that the decision to have genetic testing was the right decision. Interestingly, one participant expressed that she regretted her decision, but still felt that it was the right decision. However, most participants appeared to not be significantly impacted by this uncertainty in their daily lives and have the necessary medical and supportive care through their treating institution. This is further corroborated by the moderate “Positive Experience” scores cited on the MICRA, as the experience of genetic testing did not appear to have a significant impact on patients’ lives after disclosure. Importantly, none of our participants expressed that the choice to have genetic testing caused them a lot of harm, further supporting a streamlined model for providing accessible genetic testing to this high-risk population. 

The retrospective design of this study is a limitation, as the possibility of recall bias and differences in time from result disclosure to ascertainment may have impacted participants’ memory and response. Inherent to any retrospective study, patients who may have had a positive experience with the testing method may have been more inclined to participate in the study, introducing some potential bias in the results. Likewise, given that patients with tubo-ovarian cancers have received genetic testing exclusively through the GOIGT model since 2017, it was not feasible to have a control group of patients receiving traditional genetic counselling during the same period. Due to recall bias and the fact that many patients tested prior to GOIGT’s implementation in 2017 have passed away, it was not feasible to include a comparison group. Although introduction of a control group would not be possible with the current model at our centre, implementing a prospective study design can diminish some of this recall bias and further standardize the response time after result disclosure. 

Recruitment for this study occurred during the COVID-19 pandemic. The underlying stress associated with the pandemic may have impacted participants’ responses, especially with regards to feelings of distress or uncertainty. Comparison of results for this cohort of participants with those in the future may illustrate an artifact associated with the unanticipated circumstances. 

In this study, the limited size of the participant pool did not allow for comparisons between participants based on genetic test results (i.e., positive, negative, VUS) and the study investigator was blinded to the mutation status of all prospective participants at the time of recruitment. However, upon investigation, 12/34 study respondents tested positive, and two participants carried a variant of unknown significance (VUS), representing 41% of the respondents. This proportion is comparable if not higher than the current testing rate for this centre. Further investigations can aim to compare the experiences of participants who received positive, inconclusive, or negative test results. Similarly, comparisons between carriers of pathogenic variants in high penetrance and moderate penetrance genes may further illustrate differences in feelings of uncertainty or decision regret among participants. 

This study aimed to evaluate the experience of patients diagnosed with high-grade non-mucinous epithelial tubo-ovarian cancers who underwent genetic testing through an oncology-initiated mainstreamed genetic testing model at a tertiary healthcare centre in Montreal, Quebec. Previous evaluations of this model, as well as other international models, demonstrated an increase in genetic testing rates with a decrease in median time to results disclosure form diagnosis [11]. Participants within the current study did not appear to experience significant negative psychosocial implications from their experience with genetic testing and results disclosure and cited very low levels of decision regret. The results of this study support the continued utilization of this model at the MUHC, as well as the potential expansion of similar models for other cancer types both within our institution and throughout the province of Quebec.

## Figures and Tables

**Table 1:** Participant demographics. CEGEP: collège d’enseignement général et professionnel (general and vocational college). Education demographics denote highest level of education completed.

Participant Demographics	
Mean Age (Range)	64.62 (40–84)
Urban vs. Rural, *n* (%)	Urban: 30 (88.2)
	Rural: 4 (11.8)
Primary Language, *n* (%)	English: 20 (58.8)
	French: 12 (35.3)
	English and French: 1 (2.9)
	Other: 1 (2.9)
Work Status, *n* (%)	Retired: 17 (50.0)
	Disability/Sick Leave: 5 (14.7)
	Full-Time: 4 (11.8)
	Self-Employed, Part-Time: 1 (2.9)
	Self-Employed, Full-Time: 4 (11.8)
	Homemaker: 3 (8.8)
Education, *n* (%)	Technical/Vocational/CEGEP: 11 (32.4)
	University (Undergraduate): 10 (29.4)
	University (Graduate Degree): 6 (17.6)
	High School: 6 (17.6)
	Elementary School: 1 (2.9)

**Table 2:** Summary of mean scores on MICRA questionnaire (median, range).

	Possible Score Range	Mean (Median, Range)
Distress	0–45	3.97 (1.5, 0–19)
Uncertainty	0–20	8.35 (7.5, 0–20)
Positive Experience	0–20	9.65 (9.5, 0–20)
Total MICRA Score	0–95	21.97 (20, 0–42)

## Data Availability

The datasets generated during and/or analyzed during the current study are available from the corresponding author on reasonable request.
